# Synthesis of Mn_3_O_4_-Based Aerogels and Their Lithium-Storage Abilities

**DOI:** 10.1186/s11671-015-0960-x

**Published:** 2015-06-10

**Authors:** Huang Tang, Yongxing Sui, Xiaoqin Zhu, Zhihao Bao

**Affiliations:** School of Mathematics and Physics, Jiangsu University of Technology, 1801 Zhongwu Road, Changzhou, 213001 China; Shanghai Key Laboratory of Special Artificial Microstructure Materials and Technology, School of Physics Science and Engineering, Tongji University, 1239 Siping Road, Shanghai, 200092 China

**Keywords:** Mn_3_O_4_ aerogel, Graphene nanosheet, Lithium-ion batteries

## Abstract

Mn_3_O_4_ aerogels and their graphene nanosheet (GN) composite aerogels were synthesized by a simple supercritical-ethanol process. In the process, supercritical ethanol acted as a reductant to reduce graphene oxide and MnO_2_ gels simultaneously. The synthesized aerogels consisted of 10–20 nm Mn_3_O_4_ nanocrystallites, with BET-specific surface areas around 60 m^2^/g. The performance of the aerogels as anode materials for lithium-ion batteries was also evaluated in this study. The results showed that Mn_3_O_4_ aerogels as anode materials exhibited a reversible capacity of 498.7 mAh/g after 60 charge/discharge cycles while the reversible capacity for Mn_3_O_4_/GN composite aerogels could further increase to 665 mAh/g. The mechanisms for the enhanced capacity retention could be attributed to their porous structures and improved electronic contact with GN addition. The process should also offer an effective and facile method to fabricate many other porous metal oxide/GN nanocomposites for low-cost, high-capacity, environmentally benign material for lithium-ion batteries.

**Electronic supplementary material:**

The online version of this article (doi:10.1186/s11671-015-0960-x) contains supplementary material, which is available to authorized users.

## Background

Nanostructured materials such as nanowires, nanotubes, nanosheets, and porous nanomaterials have attracted great interest in the recent years because of the novel properties from their reduced dimensionality. They are becoming increasingly important for electrochemical energy-storage applications, especially for lithium-ion batteries [1–5]. The advantages of using nanostructured materials as electrodes for lithium-ion batteries include short path lengths for electron and Li-ion diffusion for high power, and accommodation of the strain due to Li intercalation/extraction for good cycle life, and so on [6–8]. Aerogels are well-known nanostructured materials with very high surface areas. They have a three-dimensional network of nanosized particles surrounded by macro-, meso-, and micropores. The diffusion distances for lithium ions and electrons in the nanosized aerogel particles are expected to be shorter compared with that of the solid electrode materials. Electrolytes can also easily infiltrate the entire aerogel particles. Porous structures may accommodate the strain induced by the volume expansion during the lithiation process. The above characteristics could enhance the performance (i.e., cyclability and rate capability) of corresponding materials such as the electrodes for lithium-ion batteries. For example, V_2_O_5_ aerogels were reported to have electroactive capacities greater than polycrystalline-solid V_2_O_5_ powders [9]. Thereafter, other aerogels, including MnO_2_ and Li_x_MnO_2_ as cathode materials for lithium-ion batteries, have also been investigated [10–12]. However, to our knowledge, the successful synthesis of Mn_3_O_4_ aerogels has not been reported and their lithium-storage abilities have not been studied so far. Mn_3_O_4_ is an attractive anode material for lithium-ion batteries due to high abundance of Mn element in natural resource, low cost, and environmental benignity. Its theoretical capacity can reach as high as ~936 mAh/g. However, previous studies reported that Mn_3_O_4_ showed poor performance as anode materials for lithium batteries. At a current density of 40 mA/g, the pure solid Mn_3_O_4_ powder showed a capacity lower than 300 mAh/g, which further decreased to ~200 mAh/g after ten cycles. Even for Co-doping Mn_3_O_4_, the first discharge capacity of ~900 mAh/g could be reached; however, it also further decreased to ~400 mAh/g after ten cycles [13]. Mn_3_O_4_ nanoparticles even showed worse performance. After 10 cycles, only a capacity of ~115 mAh/g was retained. Only recently, have the capacity and rate capability of Mn_3_O_4_ been greatly improved by wiring up the Mn_3_O_4_ nanoparticles through a two-step solution-phase method [14].

In this paper, we have synthesized Mn_3_O_4_ aerogels and its graphene composite aerogels for the first time by a straightforward method, a supercritical-ethanol process. The results indicate that the supercritical-ethanol process can not only serve as a drying method to obtain the porous structure of aerogels but also reduce high-valence manganese oxide and graphene oxide (GO) simultaneously. The lithium-storage abilities of Mn_3_O_4_ aerogels were also investigated. The anode based on pure Mn_3_O_4_ aerogels exhibited an initial capacity of 1274.3 mAh/g. After 60 discharge/charge cycles, the capacity of 498.7 mAh/g was retained while the capacity increased to 665 mAh/g when graphene nanosheets (GNs) were incorporated into aerogel structure to improve their electronic contact.

## Methods

### Synthesis of GO

GO was prepared by a modified Hummers method [15, 16]. Briefly, 1 g-powdered flake graphite (500 mesh) and 0.75 g of NaNO_3_ were placed in a flask. 75 mL of H_2_SO_4_ (98 wt %) was then added with mechanical stirring in an ice-water bath. After 10 min, 4.5 g of KMnO_4_ was added gradually in the flask in 1 h. After the mixture was stirred vigorously for 5 days at room temperature, 3 mL of H_2_O_2_ (30 wt % aqueous solution) was added, and the mixture was stirred for 2 h at room temperature. The mixture was washed thoroughly with a mixed aqueous solution of 3 wt % H_2_SO_4_/0.5 wt % H_2_O_2_ to remove the excess manganate and the sulfate. Then, the solution was subjected to dialysis for 3–4 days to completely remove residual metal ions and acids. A typical AFM of GO is shown in Additional file 1: Figure S1.

### Synthesis of Mn_3_O_4_-Based Aerogels

MnO_2_ wet gels were prepared by the previously reported method [17]. Briefly, 0.948 g of KMnO_4_ was dissolved in 12.5-ml de-ion water. 0.232 fumaric acid was then added into the vigorously stirred KMnO_4_ solution. The resulting brown sol was then poured into polypropylene molds for gelation and aging for 24 h. The gel then was rinsed in 1-M sulfuric acid, followed by multiple rinses with water to remove impurity. MnO_2_/GO composite was prepared by adding GO into MnO_2_ sol first. The rest of the gelation and aging procedures were the same. Wet gels were then rinsed with ethanol for several times. The rinsed MnO_2_ gels and its graphene nanosheet (GN) hybrid were subjected to a supercritical-ethanol process at 260 °C at 10 MPa for 6 h to form Mn_3_O_4_ aerogels and Mn_3_O_4_/GN composite aerogels.

### Characterization

X-ray diffraction (XRD) patterns were obtained by an X-ray diffractometer (Rigaku D/Max-RB) with high intensity Cu Kα radiation (λ = 1.5418 Å, 40 kV, 100 mA).The morphology of samples was observed by a scanning electron microscopy (SEM, JSM6700F). Transmission electron microscopy (TEM) was conducted with a JEOL JEM-2010 electron microscope operating at 200 keV. The GN content in the Mn_3_O_4_/GN composite was determined by a thermogravimeter (TG, SDT Q600), and the measurements were carried out in air over a temperature range of 30–500 °C with a ramp rate of 10 °C /min. X-ray photoelectron spectroscopy (XPS) experiments were carried out on a RBD-upgraded PHI-5000C ESCA system (Perkin Elmer) with Al Kα radiation (1486.6 eV). XPS Peak Version 4.1 software was used to perform curve fitting. The N_2_ absorption-desorption analysis was conducted on an Autosorb-1 instrument. The distribution of pore size was calculated from the desorption data using the Barret-Joyner-Halenda (BJH) method.

### Electrochemical Measurement

The powder of Mn_3_O_4_ aerogels and Mn_3_O_4_/GN composite aerogels as active materials, Super P carbon black and polyvinyldifluoride (weight ratio 80:10:10), were mixed in N-Methylpyrrolidone (NMP) solvent to produce an electrode slurry. The slurry was coated onto a copper foil using the doctor-blading method and then dried to form the working electrode. The electrochemical tests were performed using two-electrode coin-type cells with lithium as both the counter and reference electrode. 1 M of LiPF_6_ in a 1:1:1 (volume ratio) mixture of ethylene carbonate and diethyl carbonate and dimethyl carbonate was used as the electrolyte. Cell assembling was carried out in an argon-filled glove box. Galvanostatic charge-discharge cycling was conducted using a battery tester (Land, CT2100A) with a voltage window of 3–0.01 V at the current density of 50 mA/g.

## Results and Discussion

### Synthesis and Characterization of Mn_3_O_4_ and Mn_3_O_4_/GN Composite Aerogels

**Fig. 1:**
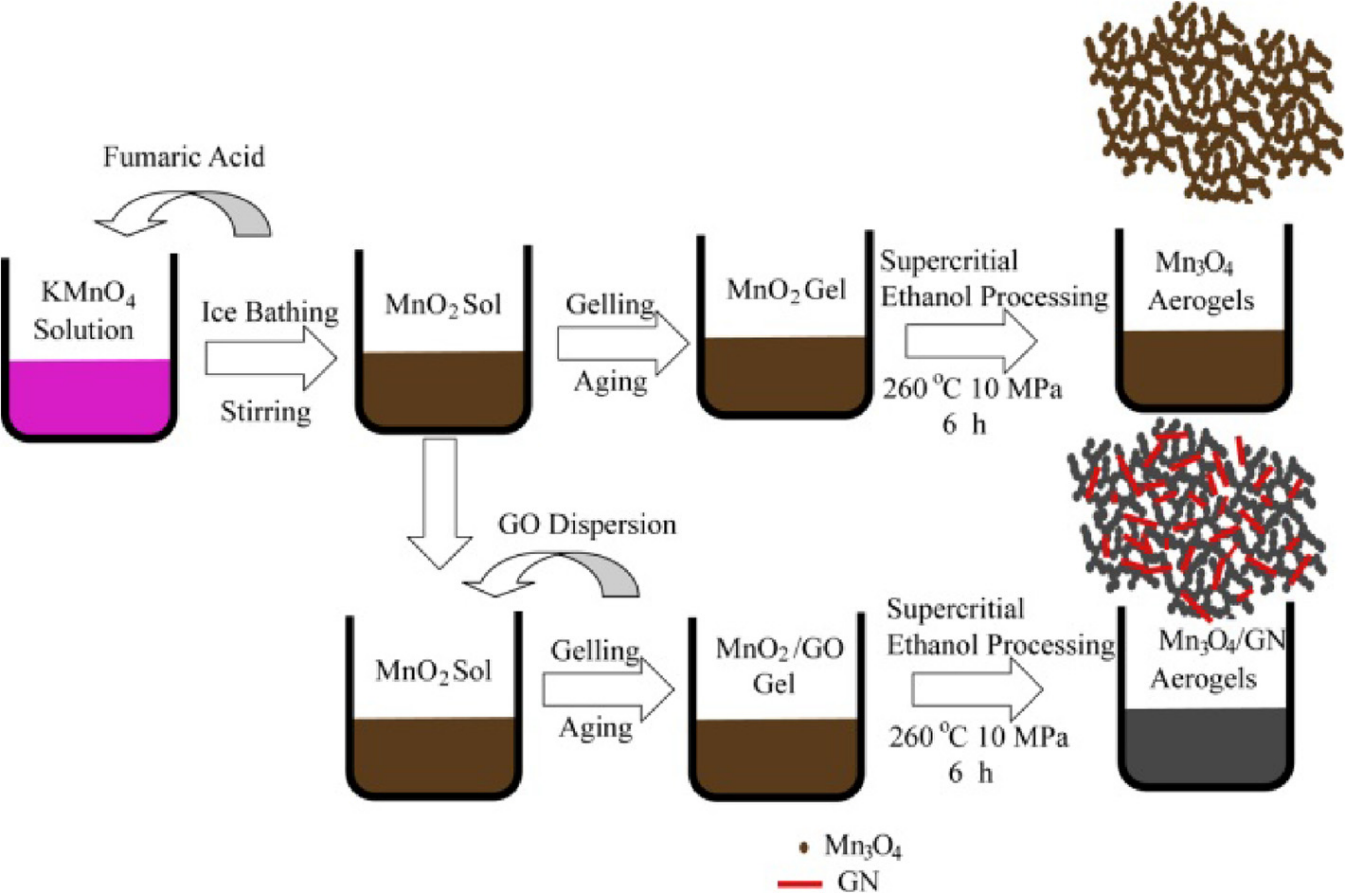
Synthetic procedure for the preparation of Mn_3_O_4_ and Mn_3_O_4_/GN aerogels

**Fig. 2:**
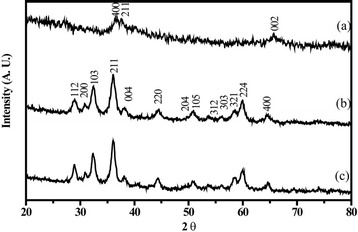
X-ray diffraction patterns of MnO_2_ dried gel **a**, Mn_3_O_4_ aerogels **b**, and Mn_3_O_4_/GN composite aerogels **c**

The process for preparing Mn_3_O_4_ and Mn_3_O_4_/GN aerogels is illustrated schematically in Fig. 1. MnO_2_ gels were prepared by adding fumaric acid to a stirred KMnO_4_ solution, following the reaction below [18, 19]:

$$ 3\mathrm{HOOCHC} = \mathrm{CHCOOH} + 10{\mathrm{MnO}}_{4^{-}} + 10{\mathrm{H}}_3{\mathrm{O}}^{+}\to 10{\mathrm{MnO}}_2+18{\mathrm{H}}_2\mathrm{O} + 6{\mathrm{C}\mathrm{O}}_2\uparrow +3{\mathrm{H}}_2{\mathrm{C}}_2{\mathrm{O}}_4 $$

as synthesized monoliths of Mn_3_O_4_ gels and Mn_3_O_4_/GN composite gels after the supercritical-ethanol process are brown and gray, respectively (Additional file 1: Figure S2). Figure 2a shows the XRD pattern of MnO_2_ dried gel sample. The broad-peak profile indicates its amorphous nature. Broad peaks at 2θ = 36.6°, 37.5° and 65.8° can be indexed to α-MnO_2_ (JCPDS No. 44–0141). After the supercritical-ethanol process (260 °C, 10 MPa), plenty of sharp peaks appear (Fig. 2b and c) and fit very well with the XRD pattern of Mn_3_O_4_ (JCPDS No. 24–0734), indicating formation of Hausmannite-type Mn_3_O_4_. No peaks of any other phases or impurities are observed, demonstrating that high-purity Mn_3_O_4_ can be obtained using the above method. According to Scherrier analysis, the size of Mn_3_O_4_ is estimated to be ~10 nm. The above results indicate that MnO_2_ was reduced into Mn_3_O_4_. The following chemical reaction was suggested to happen in the supercritical-ethanol process,

$$ 3\mathrm{M}\mathrm{n}{\mathrm{O}}_2\left(\mathrm{s}\right)+2{\mathrm{C}}_2{\mathrm{H}}_5\mathrm{O}\mathrm{H}\kern0.5em \left(\mathrm{l}\right)\to \mathrm{M}{\mathrm{n}}_3{\mathrm{O}}_4\left(\mathrm{s}\right)+2\mathrm{C}{\mathrm{H}}_3\mathrm{C}\mathrm{O}\mathrm{H}\ (1)+2{\mathrm{H}}_2\mathrm{O}\;\left(\mathrm{l}\right) $$

Supercritical ethanol acted as the reducing agent to reduce MnO_2_ into Mn_3_O_4_ during the process [20, 21]. Meanwhile, high pressure and high temperature facilitated the crystallization of Mn_3_O_4_.

**Fig. 3:**
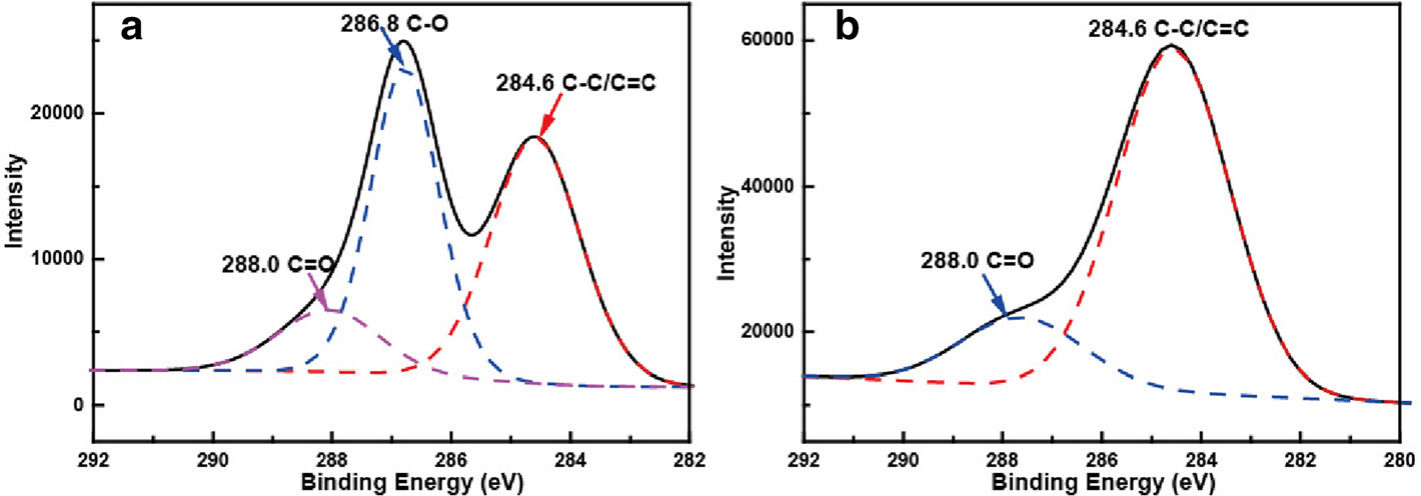
XPS of graphene oxide **a** before and **b** after the supercritical-ethanol process

**Fig. 4:**
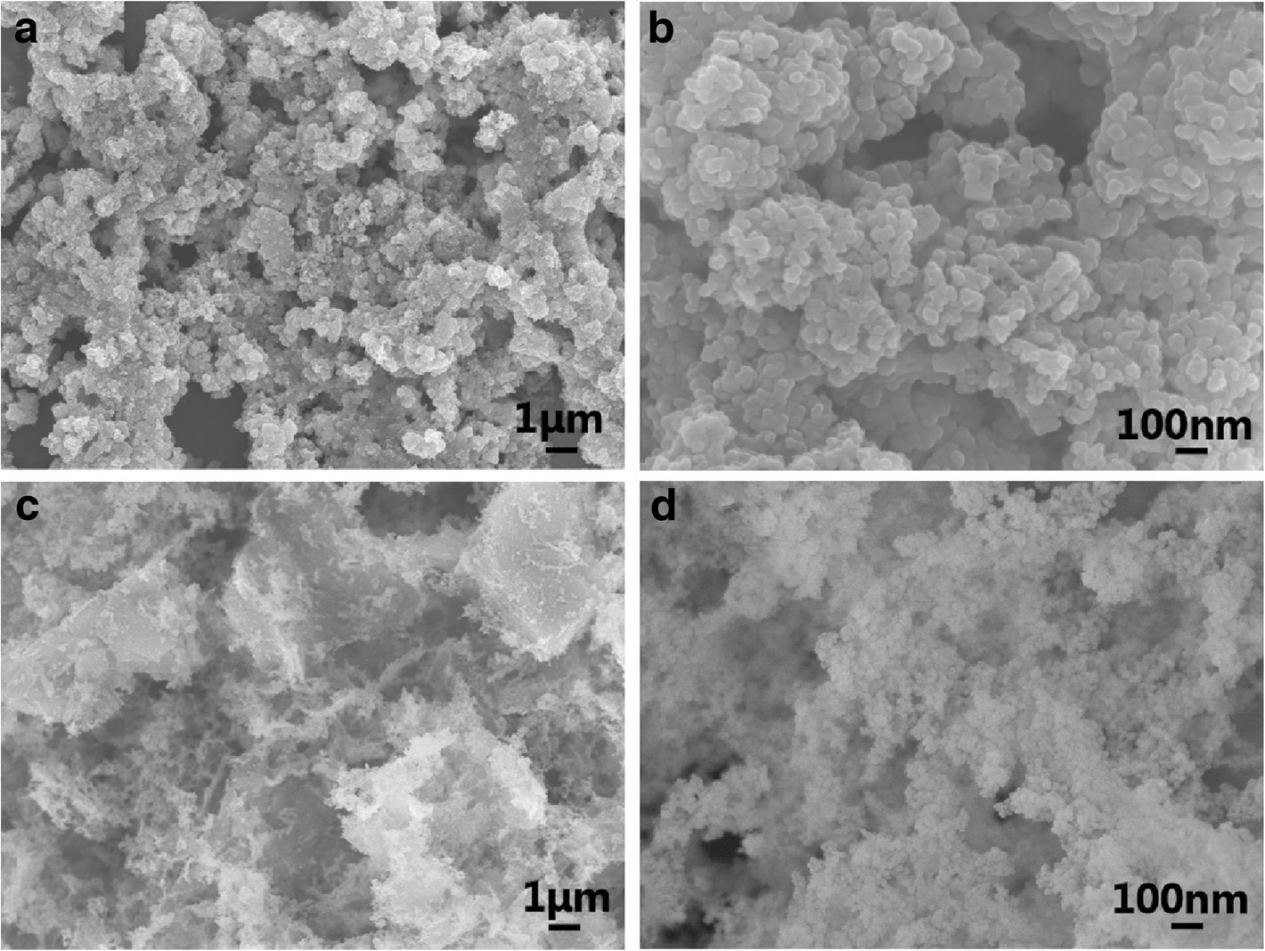
SEM images of Mn_3_O_4_ aerogel particles and Mn_3_O_4_/GN composite aerogel particles: **a** low magnification image of the Mn_3_O_4_ aerogel particles; **b** high magnification of **a**; **c** low magnification image of the Mn_3_O_4_/GN composite aerogel particles; **d** high magnification of **c**

At the same time, alcohols were reported as effective reductants to reduce GO into highly conductive GN [22, 23]. Thus, supercritical ethanol under high temperature and high pressure was expected to have improved reducibility to reduce GO pre-mixed in MnO_2_ gels into GN. Here, XPS (Fig. 3) was used to analyze GO powder and GO in composite after the supercritical-ethanol process. Curve fitting of the spectra was performed using Gaussian-Lorentzian peak shape after a Shirley background correction. For GO, three peaks located at 284.6, 286.8, and 288.0 eV could be assigned to the C-C/C = C, C-O, and C = O species, respectively [24, 25]. The peak at 286.8 eV after supercritical-ethanol treatment almost disappeared, indicating that the C-O species were removed by the supercritical-ethanol process. Hydrogenating capacity of ethanol under the supercritical condition was responsible for the partial elimination of oxygen-containing functional groups on GO. The GN content in the Mn_3_O_4_/GN nanocomposite was quantitatively determined to be 7.3 wt % by thermogravimetric analysis (Additional file 1: Figure S3). Figure 4 shows SEM images of the Mn_3_O_4_ aerogels and Mn_3_O_4_/GN composite aerogels. Samples exhibited porous structures composed of 10–20 nm Mn_3_O_4_ nanoparticles. In Mn_3_O_4_/GN composite aerogels, the micron-sized reduced GO sheets randomly distributed, and a lot of Mn_3_O_4_ nanoparticles were attached on their surface. Due to its good solubility in the water solution, GO could be dispersed in the gels very well. Such GO was frozen in the gel structure and kept with good dispersion during the subsequent reduction in the supercritical-ethanol process. The process somewhat avoided the agglomeration problem, which usually accompanied the reduction of GO into GN in the solution.

**Fig. 5:**
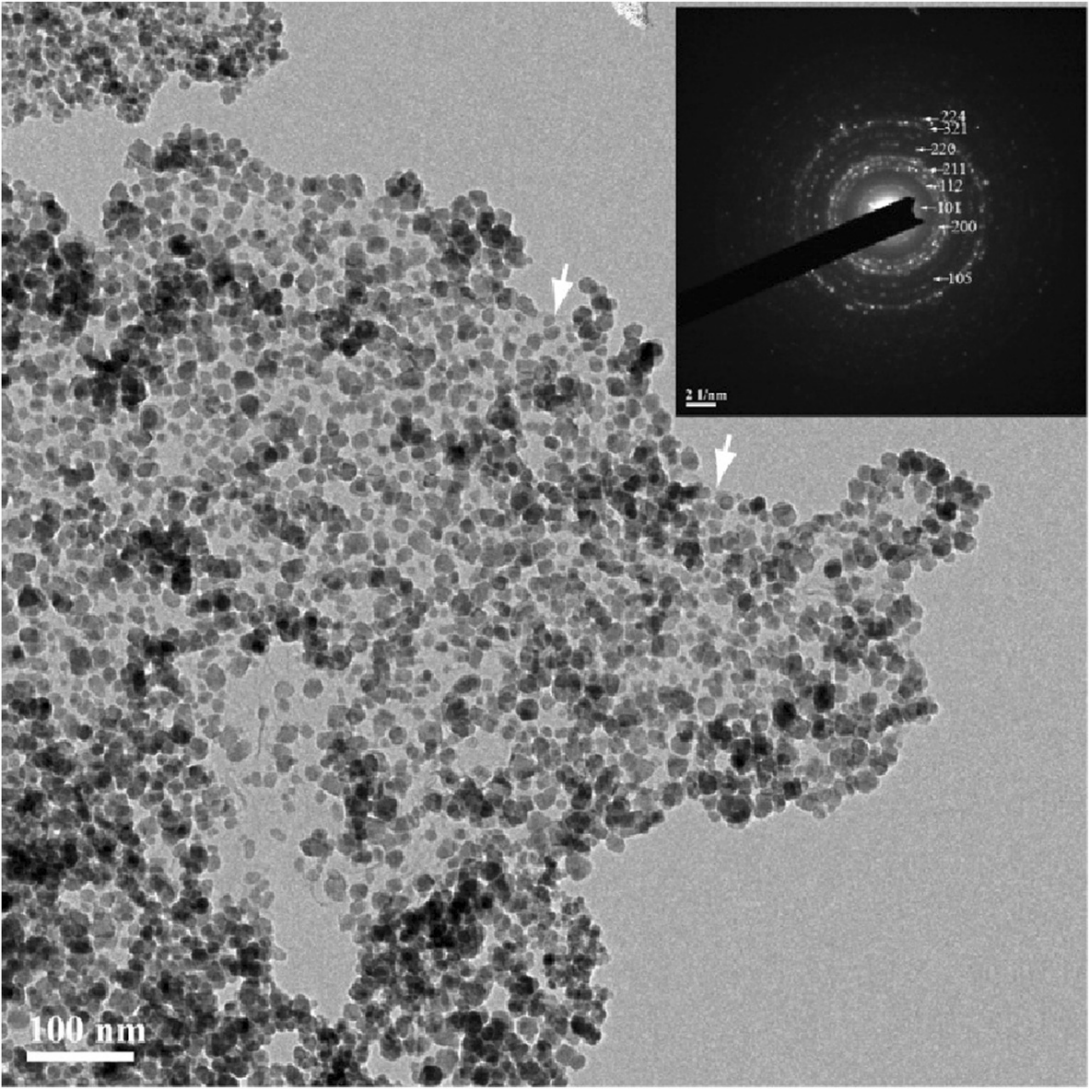
Typical TEM image of Mn_3_O_4_/GN composite aerogels and their SAED pattern (*inset*). *White arrows* indicate the edges of GN

The bright field TEM image (Fig. 5) of the resulting Mn_3_O_4_/GN nanocomposite products confirms that the 10–20 nm Mn_3_O_4_ nanoparticles decorated on the surface of GN. A selected area electron diffraction (SAED) pattern of the final products is shown in the insert of Fig. 5. All the concentric diffraction rings are related to the phase of Mn_3_O_4_, consistent with the relevant XRD analyses. Surface area, pore size, and pore volume are important characteristics for aerogel materials. The data related with Mn_3_O_4_ aerogels and Mn_3_O_4_/GN aerogels are summarized in Table 1. Nitrogen adsorption/desorption isotherms (Additional file 1: Figure S4) of the Mn_3_O_4_ aerogels and Mn_3_O_4_/GN composite aerogels are type IV isotherms with H1 hysteresis loops, which are characteristic of an interconnected mesoporous system with cylindrical pores. BET-specific surface areas are 69 and 67 m^2^/g, respectively. The BET-specific areas are not as high as the reported value (~200 m^2^/g) of MnO_2_ aerogels [17], which might be due to crystallization and coarsening of Mn_3_O_4_ in the supercritical-ethanol process at relative high temperature and pressure.

**Table 1:** Summary of N_2_ adsorption/desorption data for Mn_3_O_4_ aerogels and Mn_3_O_4_/GN composite aerogels

Sample	Specific surface area	Pore volume	Average pore diameter
	(m^2^/g)	(cm^3^/g)	(nm)
Mn_3_O_4_ aerogels	69	0.25	14.5
Mn_3_O_4_/GN aerogels	67	0.27	16.1

### Electrochemical Properties of Anodes Based on Mn_3_O_4_ Aerogels and Mn_3_O_4_/GN Composite Aerogels

**Fig. 6:**
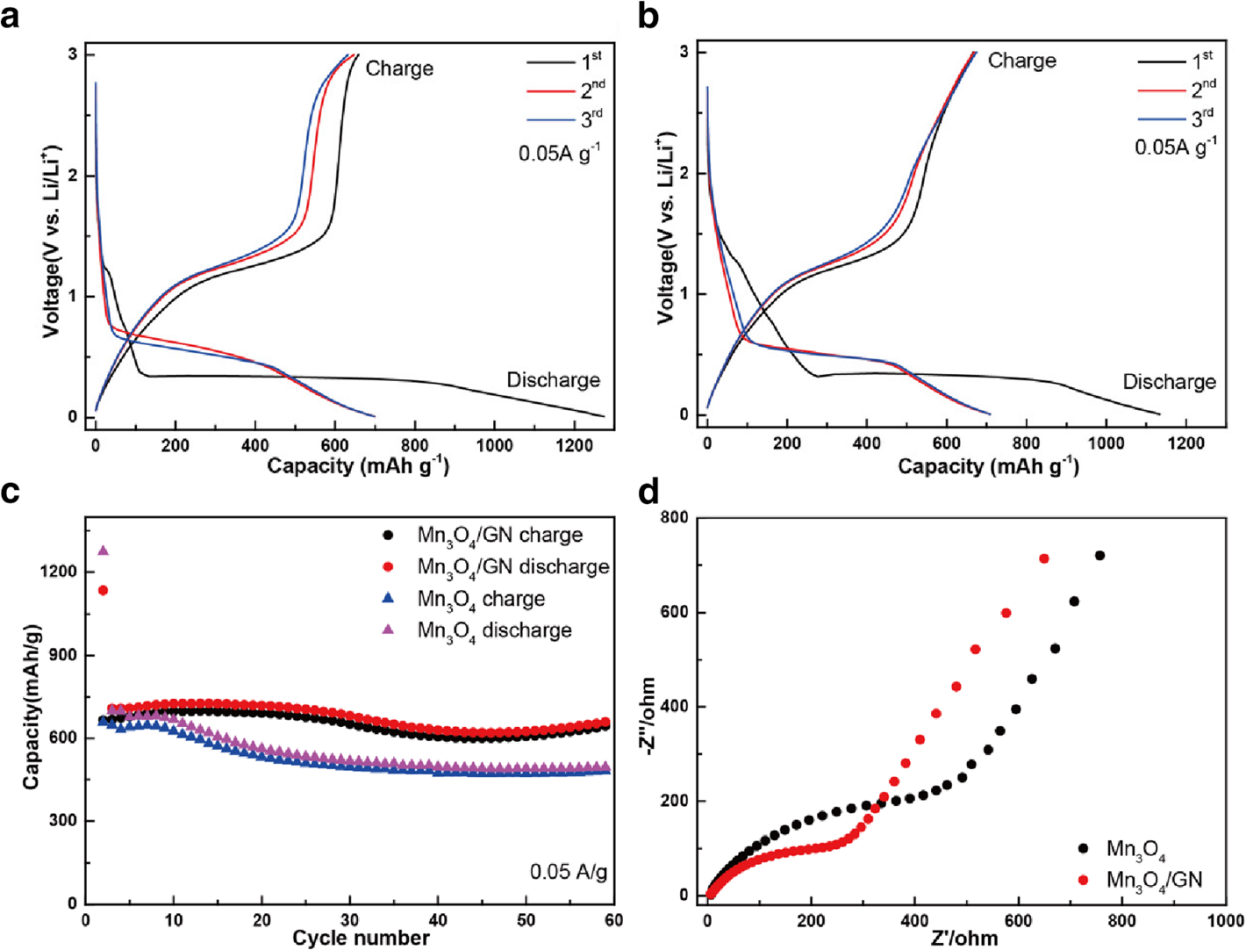
Galvanostatic charge-discharge voltage profiles of anodes based on Mn_3_O_4_ aerogels **a** and Mn_3_O_4_/GN composite aerogels **b**, cycle performance of Mn_3_O_4_ aerogels and Mn_3_O_4_/GN composite aerogels **c**, and Nyquist plots of the Mn_3_O_4_ and Mn_3_O_4_/GN electrodes **d**

To evaluate the electrochemical performance of Mn_3_O_4_-based aerogels, the samples were used as the anodes for Li-ion battery. Figure 6a,b show the charge and discharge curves for anodes based on Mn_3_O_4_ and Mn_3_O_4_/GN aerogels, respectively. In the first discharge curve, a sloping plateau in the range of ~1.5– 0.3 V is observed. It might be due to the formation of solid-electrolyte interface (SEI) film on the active materials. The well-defined voltage plateau around 0.35 V reflected the reduction reaction of Mn_3_O_4_ as follows: Mn_3_O_4_ + 8Li^+^ + 8e^−^ → 3Mn(0) + 4Li_2_O [26]. The charge curve shows a plateau at ~1.2 V due to the reverse reaction [14]. Figure 6c compares the cycle performance of Mn_3_O_4_ electrode and Mn_3_O_4_/GN electrode at a current of 50 mA/g. The Mn_3_O_4_ electrode shows an initial discharge capacity of 1274.3 mAh/g with an initial Coulombic efficiency of 51.7 %. With GN addition, the initial discharge capacity for Mn_3_O_4_/GN was measured to be 1134.2 mAh/g with an initial Coulombic efficiency of 58.7 %. The capacity loss results from the formation of SEI film, some other irreversible side-reactions, and the disengagement of the Mn_3_O_4_ particles from the electrodes. After 60 cycles, the Mn_3_O_4_/GN composite electrode retained a discharge capacity of 665 mAh/g with a Coulombic efficiency of 97.7 %, compared with the Mn_3_O_4_ electrode with a discharge capacity of 498.7 mAh/g with a Coulombic efficiency of 97.2 %. The specific capacities of Mn_3_O_4_ and Mn_3_O_4_/GN are both much higher than the reported values of the anode based on Mn_3_O_4_ nanoparticles, 115 mAh/g after ten cycles [14]. The improved electrochemical performance of Mn_3_O_4_ aerogels could be attributed to the structure of aerogels. The high porosity from mesopores of aerogel structures facilitates the transportation of lithium ions in the aerogel particle, and the large surface area of aerogel enhances the utilization of active materials [27]. Further improvement of electrochemical properties of Mn_3_O_4_/GN can be attributed to the wiring effect of GN, that is, that the large-sized GNs can build an excellent conductive network which facilitates the electron transfer in the structure of aerogels. This was supported by electrochemical impedance spectroscopy (EIS) measurements performed after the fifth cycle. The Nyquist plots of the Mn_3_O_4_ and Mn_3_O_4_/GN electrodes are presented in Fig. 6d. Both Nyquist plots consist of one semicircle at medium-frequency region, which could be related to charge-transfer resistance [28]. The diameter of the semicircle of Mn_3_O_4_/GN electrode is smaller than that of pure a Mn_3_O_4_ electrode, indicating the enhanced electron conductivity of the Mn_3_O_4_/GN [29].

## Conclusions

Mn_3_O_4_ aerogels and its GN composite aerogels were synthesized by the simple supercritical-ethanol process on MnO_2_ and MnO_2_/GO gels, respectively. Supercritical ethanol served as both drying and reducing agents of MnO_2_ and GO in the process. The samples were characterized by X-ray, SEM, XPS, and TEM. The electrochemical measurements demonstrated that Mn_3_O_4_ aerogels possessed better capacity retention compared with Mn_3_O_4_ nanoparticles. The enhanced performance of the synthesized Mn_3_O_4_ was ascribed to its porous aerogel structure. With GN addition to Mn_3_O_4_ aerogels, their electrochemical property was improved further. The higher capacity retention was attributed to the fact that GNs enhanced the electron transfer and reduced the resistance within Mn_3_O_4_. The process should also offer an effective and convenient technique to fabricate many other metal oxide/graphene porous nanocomposites for low-cost, high-capacity, environmentally benign material for lithium-ion batteries.

## Additional file

Additional file 1:
**The AFM image of GO, the photo of Mn**_**3**_ **O**_**4**_ **aerogel monoliths, and the thermogravimetric analyses and Nitrogen adsorption/desorption isotherms of Mn**_**3**_ **O**_**4**_ **aerogel.** **Figure S1.** AFM image of GO, **Figure S2.** Photos of monoliths of Mn_3_O_4_-based aerogels, **Figure S3.** Thermogravimetric analyses of Mn_3_O_4_-based aerogels, **Figure S4.** N2 isotherms and pore size distributions of Mn_3_O_4_-based aerogels.
