# Supplementary material for: Synthesis of Mn3O4-Based Aerogels and Their Lithium-Storage Abilities
Source: Nanoscale Res Lett. 2015 Jun 10;10:260. doi: 10.1186/s11671-015-0960-x (PMC4478184; doi:10.1186/s11671-015-0960-x)
Supplement: Additional file 1: — The AFM image of GO, the photo of Mn 3 O 4 aerogel monoliths, and the thermogravimetric analyses and Nitrogen adsorption/desorption isotherms of Mn 3 O 4 aerogel. Figure S1. AFM image of GO, Figure S2. Photos of monoliths of Mn3O4-based aerogels, Figure S3. Thermogravimetric analyses of Mn3O4-based aerogels, Figure S4. N2 isotherms and pore size distributions of Mn3O4-based aerogels. [file 11671_2015_960_MOESM1_ESM.doc]

**Additional file**


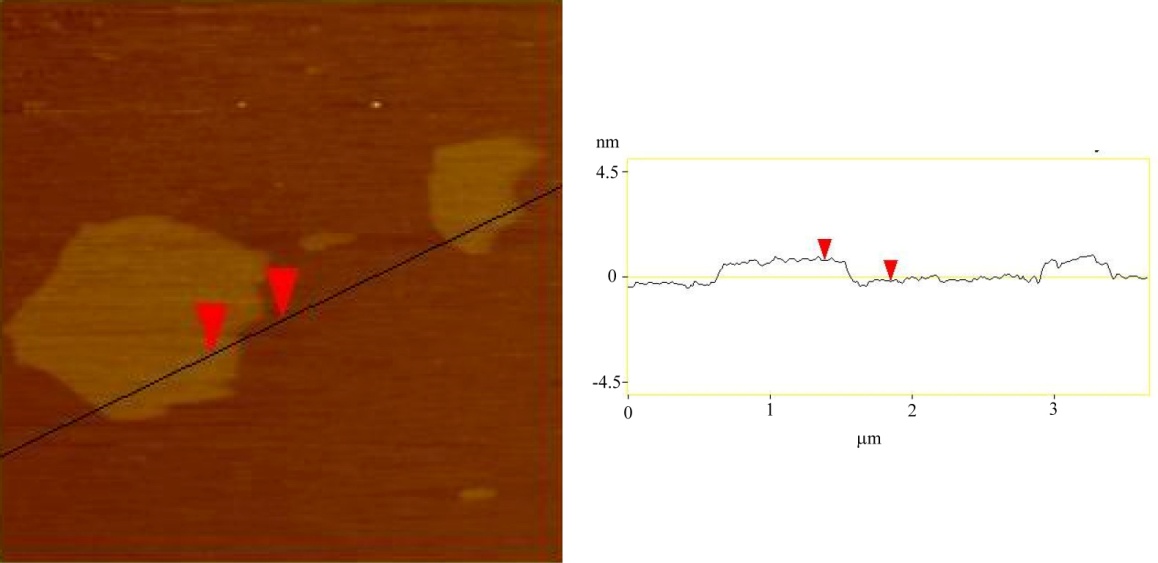


Figure S1. A typical AFM image of GO nanosheets with height of ~0.883 nm.


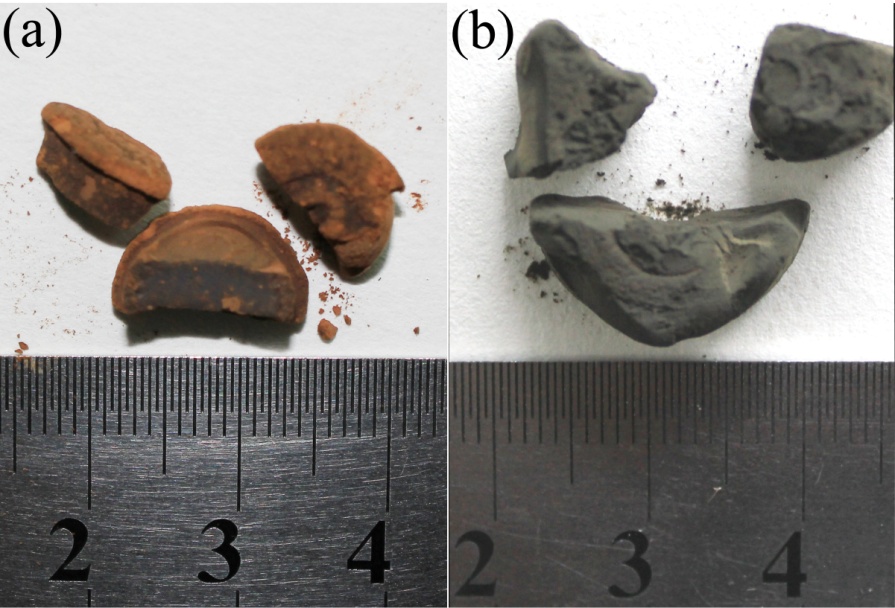


Figure S2. Photos of Mn_3_O_4_ aerogel monoliths (a) and Mn_3_O_4_/GN composite aerogel monoliths.

Figure S3. Thermogravimetric analyses of Mn_3_O_4_-based aerogels


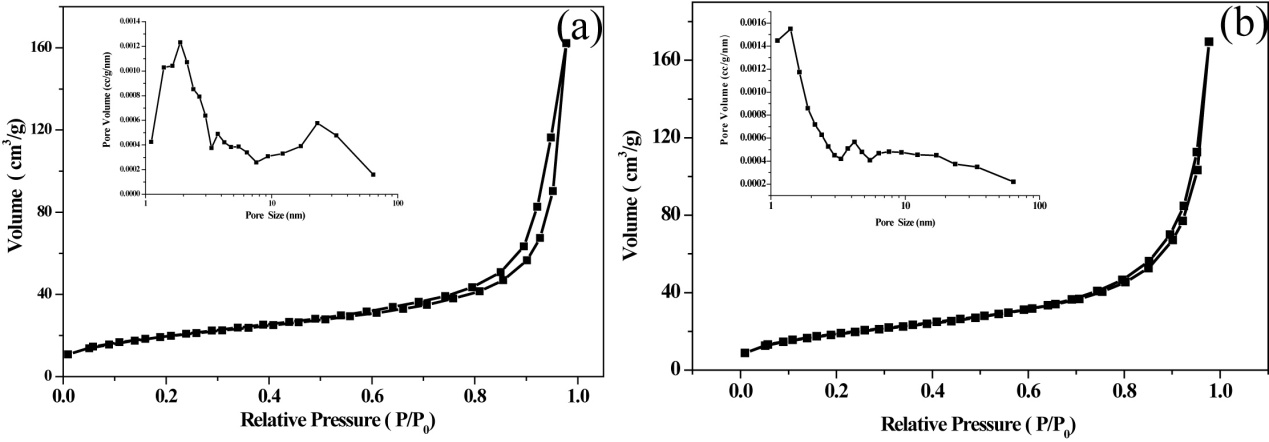


Figure S4. Nitrogen adsorption/desorption isotherms of Mn_3_O_4_ aerogels (a) and Mn_3_O_4_/GN composite aerogels (b). Inserts are pore size distributions of the corresponding aerogels.
